# Is rest-activity rhythm prospectively associated with all-cause mortality in older people regardless of sleep and physical activity level? The ‘Como Vai?’ Cohort study

**DOI:** 10.1371/journal.pone.0298031

**Published:** 2024-02-16

**Authors:** Andrea Wendt, Renata Moraes Bielemann, Fernando C. Wehrmeister, Luiza I. C. Ricardo, Werner de Andrade Müller, Adriana Kramer Fiala Machado, Maurício Feijó da Cruz, Andréa D. Bertoldi, Soren Brage, Ulf Ekelund, Luciana Tovo-Rodrigues, Inácio Crochemore-Silva

**Affiliations:** 1 Graduate Program in Health Technology, Pontifícia Universidade Católica do Paraná, Curitiba, Brazil; 2 Post-Graduation Program in Epidemiology, Federal University of Pelotas, Pelotas, Brazil; 3 School of Nutrition, Federal University of Pelotas, Pelotas, Brazil; 4 Medical Research Council Epidemiology Unit, University of Cambridge, Cambridge, United Kingdom; 5 Federal University of Pelotas, Pelotas, Brazil; 6 Department of Sports Medicine, Norwegian School of Sport Sciences, Oslo, Norway; 7 Department of Chronic diseases, Norwegian Institute of Public Health, Oslo, Norway; 8 Post-Graduation Program in Physical Education, Federal University of Pelotas, Pelotas, Brazil; Federal University of Rio Grande do Sul: Universidade Federal do Rio Grande do Sul, BRAZIL

## Abstract

**Objective:**

This study aims to test the association of rest-activity rhythm (intradaily variability and interdaily stability) with all-cause mortality in an older adult cohort in Brazil. It also assesses whether the amount of time spent at each intensity level (i.e., physical activity and nocturnal sleep) interferes with this association.

**Methods:**

This cohort study started in 2014 with older adults (≥60 years). We investigated deaths from all causes that occurred until April 2017. Rest-activity rhythm variables were obtained using accelerometry at baseline. Intradaily variability indicates higher rhythm fragmentation, while interdaily stability indicates higher rhythm stability. Cox proportional-hazard models were used to test the associations controlling for confounders.

**Results:**

Among the 1451 older adults interviewed in 2014, 965 presented valid accelerometry data. During the follow-up period, 80 individuals died. After adjusting the analysis for sociodemographic, smoking, morbidity score, and number of medicines, an increase of one standard deviation in interdaily stability decreased 26% the risk of death. The adjustment for total sleep time and inactivity did not change this association. On the other hand, the association was no longer significant after adjusting for overall physical activity and moderate to vigorous physical activity.

**Conclusion:**

Rest-activity rhythm pattern was not associated with mortality when physical activity was considered, possibly because this pattern could be driven by regular exercise. Promoting physical activity remains a relevant strategy to improve population health.

## Introduction

Population ageing is a global phenomenon defined by decreased fertility rates and increased longevity, resulting in a shift in the age pyramid for a given population [1]. Demographic changes are related to decreases in mortality rates due to infectious diseases and increases in chronic diseases [1]. Chronic diseases are partially determined by lifestyle, raising the need for special attention to healthy behaviours to improve older adults’ health. In this context, understanding the behaviours that may help older people achieve better life quality or longevity is fundamental to meet the demands of this large-sized group while avoiding the overload of health services [2, 3].

At old age, a series of physiological changes occur in the organism and, when combined with the environment, these modifications may result in health problems [4]. Among these changes, we have observed modifications in circadian rhythm, a decrease in mobility or physical activity (PA) level, as well as a reduction in sleep duration [5, 6]. Many studies have demonstrated that sleep problems and physical inactivity are associated with a wide range of health problems, including all-cause mortality in older adults [7–9]. On the other hand, circadian rhythm aspects, which consider the 24-hour patterns, are less explored in the literature.

Circadian rhythm, naturally more disrupted in advanced age, controls a series of physiological processes in the body, including the rest-activity rhythm (RAR) [6]. RAR may be defined as the daily pattern of activity (daily movement) and rest (sleep and inactivity) [10]. However, the RAR is not only a simple total amount of time spent in the aforementioned activities [10]. During a day, changes in the rest-activity cycle are expected. PA, for example, is usually based on periods of higher acceleration while inactivity time and sleep are based on periods of lower acceleration. Nevertheless, the regularity of this pattern and the dependency relationship with the total amount of time spent at each intensity level is scarcely explored in the literature.

One approach to measure RAR is non-parametric measures, such as intradaily variability and interdaily stability. The former indicates the fragmentation of rhythm in a 24-hour cycle, in other words, the frequency of changes between rest and activity during each 24-hours. The latter indicates the rhythm stability through the days, that is, how similar this 24 hour-pattern is between days [11]. These two indicators together provide a proxy of general rest-activity pattern, where high intradaily variability or low interdaily stability may indicate problems in circadian rhythm that could lead to dysfunctions in many health aspects regulated by it. For instance, higher intradaily variability and lower interdaily stability were associated with a higher risk for cognitive impairment [12]. In contrast, higher interdaily stability reduces the risk of metabolic syndrome [13] and all-cause mortality [14].

The literature already explored the isolated effects of PA [7, 9] and sleep [8] in mortality, but little is known if the more relevant aspects are in the total amount spent in each behaviour or 24h pattern. This study examines the association of intradaily variability and interdaily stability with all-cause mortality in an older adult cohort in Brazil. It further aims to verify if the associations between variability and stability are independent of PA and sleep. We hypothesize that a less fragmented and more stable rhythm will be associated with a lower risk of death regardless of PA and sleep duration.

## Material and methods

### Setting and sampling

This longitudinal analysis is part of “COMO VAI?” study (*Consórcio de Mestrado Orientado para a Valorização da Atenção ao Idoso*–“HOW ARE YOU?” Masters Consortium for Valuation of Older Care), a population-based cohort study with older adults (60 years or older). The study was carried out in Pelotas, a mid-sized city in southern Brazil (approximately 340,000 inhabitants) [15]. The baseline occurred in 2014, and participants were contacted again two years later (2016–2017).

Two sampling stages were performed to obtain a representative sample. Initially, 133 census sectors of the city were randomized out of 488 estimated by the last National Demographic Census [15] according to the mean income order. Afterwards, 31 households were systematically selected in each sector, attempting to identify at least 12 older adults per sector (0.4 per household) to reach the estimated of 3,745 households to be visited. Older adults residing in hospitals, long-term institutions, and prisons or with disabilities that could hinder the interview were excluded. Additional information is available elsewhere [7].

Trained female interviewers collected data at the participants’ households between January and August 2014 (baseline). A questionnaire investigating health aspects and specific measurements of the study were administered. Between November 2016 and April 2017, follow-up interviews were carried out by phone or household visits when participants were not found by phone. In this follow-up, personal information and vital statistics were also verified.

The “COMO VAI?” study was approved by the Research Ethics Committee of the School of Medicine of Federal University of Pelotas (CAAE: 54141716.0.0000.5317). Baseline data was collected in 2013/2014 and new follow-up was carried out in 2017. Informed consent was obtained from all participants prior to interviews in both follow-ups. Relatives or neighbors who reported deaths also signed the informed consent. Verbal consent was given when the interviews were conducted by phone. In the phone interviews, older adults were reminded about face-to-face previous contact and, when authorized, the interview was carried out. This procedure also was approved by the same Ethic Committee.

### Outcome

Mortality Information was identified using reports from relatives, neighbors, death certificates and confirmed against the Mortality Information System of the Municipal Health Service of Pelotas. Refusals or losses were considered alive due to a lack of official death information. Deaths that occurred up to April 30^th^, 2017, were recorded, and only all-cause mortality was considered in this analysis [7]. Person years were calculated from the date of interview to the date of death or loss to follow up or April 30^th^, 2017, whichever came first. Losses were considered proportional over time.

### Accelerometry

At the baseline, all participants were invited to wore a triaxial accelerometer (GENEActivity, Activinsights Ltd, Kimbolton, Cambs, UK) on their non-dominant wrist for seven days during 24 hours a day, including water-based activities. This device provides raw data expressed in gravitational equivalent units (1000 m*g* = 1 *g*). The initialization and data download were performed using GENEActivity software, and data processing was performed in R-program using the GGIR package [16]. Accelerometry data were collected using a time resolution of 87.5 Hz. Activity-related acceleration was calculated using the Euclidean Norm (vector magnitude of the three axes) minus 1 g ($ENMO=\sqrt{x^{2}+y^{2}\;+z^{2}}-1g$) and summarized in the epoch of 5 s [17]. Exclusions were carried out due to low data quality (N = 12) and individuals who provided fewer than three days’ wear-time (N = 10).

#### Rest-activity rhythm variables

RAR variables were obtained from accelerometry. The two main exposures are intradaily variability and interdaily stability. The approach used to calculate these two variables in the GGIR package follows the original work published by Van Someren et al., where the original equations and methodological details may be found [18].

Intradaily variability reflects transitions between rest and activity in 24 hours. This variable is a continuous score varying from zero to two. It may be calculated as the ratio of mean squares of the difference between all consecutive hours and mean squares around the grand mean (overall variance) [18]. Higher values in intradaily variability indicate higher rhythm fragmentation [18].

Interdaily stability indicates the rhythm similarity across the days and synchronization with *zeitgebers*, which are expected to be constant since they are environmental factors such as sunlight, eating patterns, etc. Interdaily stability can be calculated as the ratio between the average 24-hour pattern variance around the mean and the overall variance [18]. Interdaily stability varies from zero to one, with higher values indicating higher rhythm stability [18].

Both variables were standardized as z-scores to make the interpretation of the results easier.

#### Physical activity and sleep variables

We measured the following variables: total sleep time, inactivity, overall PA, and moderate-to-vigorous PA (MVPA). Physical activity/inactivity were calculated based on different thresholds for acceleration (m*g*), excluding sleep time.

The sleep detection aims to identify automatically the probable period of sleep once we have no sleep log collected [19]. We used the HDCZA algorithm, available on GGIR package and based on absence of changes in z-angle wrist rotation for each time window. For our study we are using threshold of 3º on wrist rotation and 5 minutes window size based on results of a previous study [20]. Briefly, this detection starts identifying and summarizing changes in wrist z-angle in successive windows of 5 minutes. After that, 10^th^ percentile of these values is calculated for each individual, identifying periods with lower change. Thus, blocks with duration of at least 30 minutes without changes above the threshold are kept (blocks with gaps between them higher than 60min are excluded). This results in a detection of the longest period of the day (noon to noon) with lower changes in z-angle–the probable sleep time window [19]. After that, all minutes inside this window are classified as sleep or wake (sleep = changes in z-angle below 3º) [20]. This sleep detection procedure was previously used on the research community [21–23].

These physical activity/inactivity and sleep variables were used to adjust the analysis as explained below. Definitions for each intensity level (overall PA, MVPA, inactivity, and nocturnal sleep) were the following:

- MVPA: Moderate to vigorous PA (≥100m*g*) [24] with five-minute bouts, expressed in blocks of 10 minutes per week to improve interpretation.
- Inactivity: Total minutes per week spent in inactivity (<50 m*g*) [25], expressed in blocks of 10 minutes per week to improve interpretation. This definition is usually used for sedentary behaviour but because our device does not include posture information, we opted to label this variable as inactivity.
- Overall PA: Total acceleration (ENMO metric—Euclidean norm minus 1*g*) expressed in m*g* [17].
- Total sleep time: Total of minutes classified as sleep within sleep onset and sleep end) during the night [20], expressed in blocks of 10 minutes per week to improve interpretation.

### Covariates

The following variables were used to describe the sample and for adjustments, collected at baseline assessment: sex (male/female), age (60–69; 70–79; ≥ 80 years old), years of education (none; <8 years; ≥ 8 years), smoking (never/yes/former smoker) and socioeconomic status. Socioeconomic status was based on the National Economic Index (*Índice Econômico nacional*–IEN), divided into quintiles with the first representing the poorest group and the fifth, the richest one [26]. We also used a score for morbidity and the number of medicines. The morbidity score includes hypertension, diabetes, heart problems, heart failure, stroke, asthma, bronchitis, emphysema, arthritis, Parkinson’s disease, kidney failure, hypercholesterolemia, seizure, stomach ulcer, and cancer (0–1; 2–3; 4 or more morbidities). All morbidities were self-reported by the older adults based on medical diagnosis. The number of medicines varies from 0 to 3 or more and includes psychoanalytic, psycholeptics, antiepileptics, calcium channel blockers, diuretics, muscle relaxants, and digoxin.

### Statistical analysis

Firstly, we described the total sample, individuals with valid accelerometer data (analytic sample) at baseline and death at follow-up. Covariates were presented with percentages and 95% confidence intervals (95%CI), while RAR and specific behavior variables were presented in means and 95%CI. In descriptive analysis we opted to present accelerometer variables in their original unity (total sleep time in minutes, inactivity and MVPA in minutes, overall PA in m*g*, intradaily variability, and interdaily stability in the original score).

We presented Pearson’s correlations coefficient and a scatter plot showing the distribution for each combination of RAR and intensity level variable to examine their relationship.

Cox regression was used to examine the association of intradaily variability and interdaily stability with all-cause mortality using time up to death and presenting the hazard ratio (HR) and 95%CI. The crude analysis is presented in model 1, adjustments for sociodemographic variables in model 2, and adjustments for smoking, morbidity, and medicines in model 3.

Analyses were adjusted for these variables in models 4,5,6 and 7 (sleep, inactivity, overall PA, and MVPA, respectively) to examine whether intradaily variability and interdaily stability were independent of PA intensities and sleep duration.

A formal interaction tests was performed for the association with the sex of the individuals, but we did not find any evidence of moderation, presenting the results for men and women combined.

A sensitivity analysis was performed in order to eliminate confounding by preexisting diseases. We then excluded the deaths up to the first year of follow-up, and these results are presented in the supplementary materials. All statistical analyses were carried out in Stata 16.1 using *svy* considering the complex sampling.

## Results

The original sample of the ‘COMO VAI?’ study comprises 1451 older adults. The analyses presented in this work included only older adults with valid accelerometry data, comprising 965 individuals (analytical sample). The number of deaths identified in the evaluated period was 80. No differences were identified when comparing the original and analytical samples (Table 1). Table 1 also presents the RAR variables for analytic sample and deaths. The means of intradaily variability in total sample and deaths were 0.45 and 0.49 respectively. The values for interdaily stability in analytic sample and death were 0.21 and 0.18. Overall PA and MVPA were lower in deaths (Overall PA = 14.6 m*g*; MVAP = 9.6min) than in total sample (Overall PA = 21.7m*g*; MVPA = 2.2min). Total sleep time was also lower among survivors but overlapping 95%CI.

**Table 1:** Sample distribution at baseline (total sample and accelerometry sample) and follow-up (deaths).

	All sample baseline* N = 1451	Analytical sample** N = 965	Deaths^#^ N- = 80	
	**% (95%CI)**	**% (95%CI)**	**N**	**% (95%CI)**
**Sex**				
Male	37.0 (35.0; 39.1)	37.8 (35.3; 40.3)	39	48.8 (37.7; 60.0)
Female	63.0 (60.1; 65.0)	62.2 (59.7; 64.7)	41	51.2 (40.0; 62.3)
**Age**				
60-69y	52.3 (49.1; 55.4)	51.1 (47.7; 54.4)	21	26.3 (17.2; 37.9)
70-79y	31.8 (29.3; 34.4)	34.7 (31.7; 37.8)	30	37.5 (28.6; 50.7)
80y or more	15.9 (13.9; 18.1)	14.2 (12.1; 16.7)	29	36.3 (26.4; 47.5)
**Socioeconomic status**				
1^st^ quintile	20.5 (17.4; 24.1)	19.5 (16.2; 23.4)	24	31.6 (21.9; 43.1)
2^nd^ quintile	19.6 (16.7; 22.9)	20.3 (16.9; 24.1)	13	17.1 (9.7; 28.4)
3^rd^ quintile	20.0 (17.4; 22.9)	19.6 (16.4; 23.3)	12	15.8 (8.5; 27.6)
4^th^ quintile	20.0 (17.4; 22.8)	21.0 (18.1; 24.3)	13	17.1 (9.8; 28.1)
5^th^ quintile	19.9 (16.2; 24.2)	19.5 (15.5; 24.3)	14	18.4 (10.9; 29.3)
**Years of education**				
None	13.6 (11.4; 16.2)	14.0 (11.6; 16.8)	17	21.5 (17.8; 32.0)
<8 years	54.4 (50.2; 58.5)	54.9 (50.3; 59.4)	42	53.2 (42.7; 63.3)
≥8years	31.9 (27.2; 31.1)	31.1 (26.1; 36.6)	20	25.3 (15.7; 38.1)
**Smoking**				
No/never	54.0 (51.5; 56.5)	52.7 (49.8; 55.7)	36	45.0 (35.0; 55.4)
Yes	12.6 (10.8; 14.6)	13.3 (11.1; 15.8)	13	16.3 (9.0; 27.5)
Previous smoking	33.4 (31.0; 35.9)	34.0 (31.3; 36.8)	31	38.8 (29.5; 48.9)
**Morbidity score** ***				
0–1	25.7 (23.4; 28.0)	23.5 (21.0; 26.3)	11	14.1 (7.8; 24.2)
2–3	42.8 (40.2; 45.4)	44.5 (41.3; 44.8)	29	37.2 (26.8; 48.9)
4+	31.6 (29.2; 34.0)	32.0 (28.9; 35.1)	38	48.7 (36.9; 60.7)
**Number of medicines** ^##^				
0	31.3 (28.8; 34.0)	31.3 (28.0; 34.8)	11	14.9 (8.2; 25.5)
1	37.0 (34.1; 40.0)	38.0 (34.4; 41.7)	21	28.4 (19.9; 38.8)
2	19.3 (17.1; 21.8)	18.2 (15.6; 21.1)	21	28.4 (18.7; 40.6)
3+	12.3 (10.4; 14.5)	12.5 (10.3; 15.1)	21	28.4 (18.3; 41.3)
	**Mean (95%CI)**	**Mean (95%CI)**		**Mean (95%CI)**
**Intradaily variability**	-	0.45 (0.44; 0.46)		0.49 (0.47; 0.52)
**Interdaily stability**	-	0.21 (0.21; 0.22)		0.18 (0.16; 0.19)
**Total sleep time (min)**	-	405.3 (398.9; 411.7)		432.3 (409.1; 455.5)
**Overall PA (m*g*)**	-	21.7 (21.2; 22.2)		14.6 (13.4; 15.7)
**Inactivity (min)**	-	757.1 (749.7; 764.7)		810.1 (783.2; 837.1)
**MVPA (min)**	-	9.6 (8.6; 10.6)		2.22 (1.15; 3.29)

*All interviewed older adults in 2014

**Older adults with data for accelerometry in 2014

^#^Deaths with data for accelerometry

***morbidities: hypertension, diabetes, heart problem, heart failure, stroke, asthma, bronchitis, emphysema, arthritis, Parkinson, kidney failure, hypercholesterolemia, seizure, stomach ulcer and cancer.

^##^medicines: psychoanalytics, psycholeptics, antiepileptics, calcium channel blockers, diuretics, muscle relaxants and digoxin

**Fig 1:**
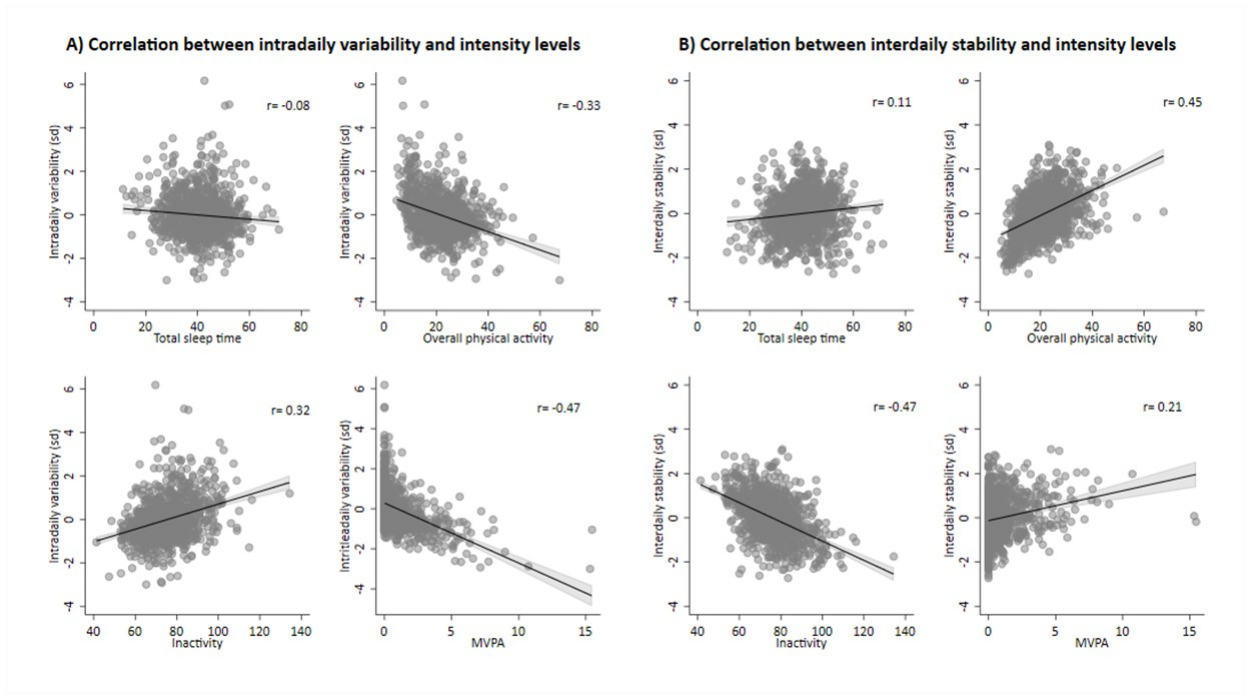
Pearson’s correlation between rest-activity variables and each intensity level variable. MVPA: Moderate to vigorous physical activity. Total sleep time, inactivity and MVPA are expressed in blocks of 10 minutes. Overall physical activity is expressed in mg.

Fig 1 shows the relationship between RAR variables and each intensity level. We observed the strongest correlation between intradaily variability and MVPA (ρ = -0.47). Considering interdaily stability, we observed the strongest correlations for interdaily stability vs. inactivity and interdaily stability vs. overall PA (ρ = -0.47 and ρ = 0.45, respectively). Total sleep time presented the weakest correlation with intradaily variability and interdaily stability data.

Supplementary material (S1 Table) presents the association of each intensity level with mortality. After adjustments, an increase of 10 minutes in total sleep time resulted in a death risk of 1.05 (95%CI = 1.02; 1.09). In the other direction, an increase of 1 m*g* in overall PA reduced the risk of death in 11% (HR = 0.89; 95%CI = 0.85; 0.95) and an increase of 10 minutes in time spent in MVPA, decreased the death risk in 54% (HR = 0.46; 95%CI = 0.25; 0.85).

Table 2 shows the crude and adjusted association between RAR variables and mortality. After adjusting for sociodemographic variables, an increase of one standard deviation in intradaily variability resulted in a higher risk of death (HR = 1.29, 95%CI:1.11; 1.50). On the other hand, an increase of one standard deviation in interdaily stability resulted in a decrease of 36% in the risk of death (HR = 0.64, 95%CI: 0.49;0.85). After the adjustments for smoking, morbidity, and the number of medicines, the association with interdaily stability variability was attenuated but remained statistically significant.

**Table 2:** Crude and adjusted association of intradaily variability and interdaily stability with all-cause mortality in older adults.

	Model 1		Model 2		Model 3	
	HR (95%CI)	P value	HR (95%CI)	P value	HR (95%CI)	P value
**Intradaily variability (sd)**	1.41 (1.22; 1.64)	<0.001	1.29 (1.11; 1.50)	0.001	1.13 (0.94; 1.35)	0.204
**Interdaily stability (sd)**	0.57 (0.44; 0.74)	<0.001	0.64 (0.49; 0.85)	0.002	0.74 (0.55; 0.98)	0.038

Model 1: Crude analysis (exposure and outcome).

Model 2: Model 1 + sex, age, socioeconomic status years of education

Model 3: Model 2 + current smoking status, morbidity score and number of medicines

Table 3 presents the association of intradaily variability and interdaily stability adjusted for each intensity level. We did not observe any association between intradaily variability and risk of death. Regarding the effect of interdaily stability on decreasing the risk of death, adjustments for total sleep time and inactivity did not alter the results (Models 4 and 5). On the other hand, the association between interdaily stability and risk of death disappeared when adjusting for overall PA and MVPA (Models 6 and 7).

**Table 3:** Association of intradaily variability and interdaily stability and all-cause mortality in older adults adjusted for intensity levels.

	Model 4		Model 5		Model 6		Model 7	
	HR (95%CI)	P value	HR (95%CI)	P value	HR (95%CI)	P value	HR (95%CI)	P value
**Intradaily variability (sd)**	1.14 (0.96; 1.36)	0.143	1.12 (0.93; 1.36)	0.236	0.92 (0.75; 1.12)	0.397	0.98 (0.79; 1.22)	0.843
**Interdaily stability (sd)**	0.72 (0.55; 0.95)	0.019	0.72 (0.52; 0.99)	0.042	1.09 (0.78; 1.51)	0.620	0.81 (0.62; 1.06)	0.117

Model 3: sex, age, socioeconomic status years of education, current smoking status, morbidity score and number of medicines

Model 4: Model 3 + adjustment for total sleep time

Model 5: Model 3 + adjustment for inactivity

Model 6: Model 3 + adjustment for overall physical activity

Model 7: Model 3 + adjustment for MVPA

Findings from sensitivity analysis (S2 and S3 Tables), excluding deaths up to one year of follow-up, are similar to main analysis. A slight reduction in HR from interdaily stability (HR _Model 3_ = 0.69, 95%CI: 0.49;0.97) was attenuated towards the null after adjusting for PA.

## Discussion

This article described the time patterning of rest-activity and examined its contribution to the association with mortality. In models adjusted for PA we did not observe any associations between rest-activity pattern and risk for mortality.

In healthy ageing, older adults often report changes in their rhythms, such as increased morning preference, earlier waking times, increased sleep latency, and decreased total sleep time [27]. Older adults demonstrate lower amplitude and more fragmented rhythm [28, 29]. Older adults become less active due to behaviour changes at advanced ages [30], and spend more time lying down during the supposed active phase of the day, which can modify their RAR [31]. Furthermore, the more fragmented rhythm found in this age group could result from chronic diseases, such as diabetes [32].

Our first level of adjustments (socioeconomic, demographic, smoking, morbidity, and medicines) showed that lower stability was associated with a higher mortality risk while fragmentation (intradaily variability) was not associated with it. Similar results were found in a prospective study conducted in Rotterdam with individuals older than 45 years, where more fragmented and less stable 24-hour activity rhythms were associated with a 20% increase in all-cause mortality risk [14]. RAR is endogenously generated by the suprachiasmatic nucleus of the hypothalamus (SCN), which sends temporal information to the entire organism through humoral and nervous signals [31]. A possible mechanism that may explain how a disrupted RAR could increase mortality risk is that the SCN is susceptible to age-related impairment due to neuronal degeneration, a lower release of arginine vasopressin, and a reduction in the amplitude of electrical activity circadian rhythms [33–35]. These alterations could reflect in a more fragmented 24-hour activity rhythm, as well as an earlier drop in temperature and onset of the melatonin phase [36]. This temporal disorganization could lead to suboptimal physiological functioning, leading to a higher susceptibility to disease [14]. In addition, RAR could not be purely biological. By maintaining regular habits such as exercise, sleep, meals, work time, etc. one could help in the organization of this rhythm. Furthermore, changes in RAR have been pointed out as useful clinical markers once disturbances in this cycle are associated with the occurrence of cognitive disfunction, dementia, cardiovascular disease, cerebrovascular changes, diabetes, and depression [37].

Our additional analyses aimed to understand if the association between RAR and mortality remains after adjustments for total time spent at each intensity level. Results showed that lower interdaily stability remains associated with all-cause mortality even after adjustments for sleep or inactivity. On the other hand, when we adjusted the analysis for overall PA and MVPA, the effect of rhythm variables on the outcome lost statistical significance. It is important to note that PA has a strong effect on reducing mortality risk, which has been already established in the literature [7, 9], and it is confirmed in our supplementary analysis. In addition, our correlation analysis exploring the relationship between intensity levels and rest-rhythm activity showed important evidence that rhythm is not independent of overall PA or time spent in MVPA. This finding suggests a relevant role of time spent in exercise activities driving the pattern of daily activities (rhythm).

In terms of biological mechanisms, the literature suggests that PA regulates the circadian rhythm, such as skeletal muscle clock modulation, thermoregulation, and hormonal secretion in response to exercise [38, 39]. In behavioural terms, sustaining a routine with regular exercise and sleep timing could improve RAR, and those with healthy habits also spent more time in PA [40]. It is difficult to establish how the hierarchical order of PA, sleep, and RAR affects mortality. Although PA could be a regulator for both sleep and RAR, adjustments for exercise variables could attenuate or even invalidate the effects of sleep or RAR on the outcome [41]. In addition, more studies remain necessary to explore the mechanisms of the association with mortality due to the complexity of the relationship between intensity levels and RAR.

We need to highlight some limitations of our study. First, the ‘COMO VAI?’ study was not designed as a cohort; therefore, typing errors in names, phone numbers, and addresses in the baseline contributed to follow-up losses and hampered the search for vital statistical information. However, double-checking of deaths through the Mortality Information System and confirmation by death certificates at the participants’ households reduced the effects of possible inconsistencies. Second, the short gap between follow-ups and the small number of deaths analyzed might have resulted in lower statistical power. Third, survival bias is a possibility because individuals with low PA levels or fragmented and unstable RAR could die before the beginning of study weakening the association. Forth, circadian cycle could be influenced by feeding time and light exposure and our study did not collect these aspects. Finally, although 33% of the original sample did not provide accelerometry data, the comparison between the analyzed and original sample did not present any differences.

Our study also presents several strengths. To the best of our knowledge, this study is the first investigation of RAR patterns using objective measurements (accelerometers) and their effect on the risk of all-cause mortality among older adults in a lower-middle-income country. Second, it is amongst the few studies assessing the effect of RAR adjusting for PA and sleep, and our results showed that it is a relevant approach due to the lack of association after PA adjustments. Lastly, it is a population-based survey of older people. We emphasize the high response rate, given the circumstances mentioned, and the high methodological quality of a representative sample.

## Conclusions

Based on our findings, RAR patterns are not associated with an increased risk of death when PA is taken into account, possibly because this pattern could be driven by regular exercise [9, 41, 42]. Promoting PA remains a relevant health strategy.

## Supporting information

S1 Table Crude and adjusted association of total sleep time, overall physical activity, time spent in inactivity and moderate-to vigorous physical activity with all-cause mortality in older adults. Model 1: Crude analysis (exposure and outcome); Model 2: Model 1 + sex, age, socioeconomic status years of education; Model 3: Model 2 + current smoking status, morbidity score and number of medicines; Total sleep time, inactivity and MVPA expressed in blocks of 10 minutes. Overall PA expressed in mg.
(PDF)

S2 Table Crude and adjusted association of intradaily variability and interdaily stability with all-cause mortality in older adults—Excluding individuals who died in the period up to one year from the baseline. Model 1: Crude analysis (exposure and outcome); Model 2: Model 1 + sex, age, socioeconomic status years of education; Model 3: Model 2 + current smoking status, morbidity score and number of medicines.
(PDF)

S3 Table Association of intradaily variability and interdaily stability and all-cause mortality in older adults adjusted for intensity levels—Excluding individuals who died in the period up to one year from the baseline. Model 3: sex, age, socioeconomic status years of education, current smoking status, morbidity score and number of medicines; Model 4: Model 3 + adjustment for total sleep time; Model 5: Model 3 + adjustment for inactivity; Model 6: Model 3 + adjustment for overall physical activity; Model 7: Model 3 + adjustment for MVPA.
(PDF)

## References

[1] Bloom DE, Luca DL. The Global Demography of Aging: Facts, Explanations, Future [Internet]. Rochester, NY; 2016 [cited 2023 Nov 7]. https://papers.ssrn.com/abstract=2834213

[2] Dominick KL, Ahern FM, Gold CH, Heller DA. Relationship of health-related quality of life to health care utilization and mortality among older adults. Aging Clin Exp Res. 2002;14:499–508. doi: 10.1007/BF03327351 12674491

[3] Achutti A, Azambuja MIR. Doenças crônicas não-transmissíveis no Brasil: repercussões do modelo de atenção à saúde sobre a seguridade social. Ciênc Saúde Coletiva. 2004;9:833–40.

[4] Scott-Warren V, Maguire S. Physiology of ageing. Anaesth Intensive Care Med. 2017;18:52–4.

[5] Duffy JF, Zitting K-M, Chinoy ED. Aging and Circadian Rhythms. Sleep Med Clin. 2015;10:423–34. doi: 10.1016/j.jsmc.2015.08.002 26568120 PMC4648699

[6] Hood S, Amir S. The aging clock: circadian rhythms and later life. J Clin Invest. 2017;127:437–46. doi: 10.1172/JCI90328 28145903 PMC5272178

[7] Bielemann RM, LaCroix AZ, Bertoldi AD, Tomasi E, Demarco FF, Gonzalez MC, et al. Objectively Measured Physical Activity Reduces the Risk of Mortality among Brazilian Older Adults. J Am Geriatr Soc. 2020;68:137–46. doi: 10.1111/jgs.16180 31592540

[8] Cappuccio FP, D’Elia L, Strazzullo P, Miller MA. Sleep duration and all-cause mortality: a systematic review and meta-analysis of prospective studies. Sleep. 2010;33:585–92. doi: 10.1093/sleep/33.5.585 20469800 PMC2864873

[9] Mok A, Khaw K-T, Luben R, Wareham N, Brage S. Physical activity trajectories and mortality: population based cohort study. BMJ. 2019;365:l2323. doi: 10.1136/bmj.l2323 31243014 PMC6592407

[10] Smagula SF. Opportunities for clinical applications of rest-activity rhythms in detecting and preventing mood disorders. Curr Opin Psychiatry. 2016;29:389–96. doi: 10.1097/YCO.0000000000000283 27636598 PMC5389454

[11] Quante M, Mariani S, Weng J, Marinac CR, Kaplan ER, Rueschman M, et al. Zeitgebers and their association with rest-activity patterns. Chronobiol Int. 2019;36:203–13. doi: 10.1080/07420528.2018.1527347 30365354 PMC6492024

[12] Smagula SF, Gujral S, Capps CS, Krafty RT. A Systematic Review of Evidence for a Role of Rest-Activity Rhythms in Dementia. Front Psychiatry. 2019;10:778. doi: 10.3389/fpsyt.2019.00778 31736798 PMC6832024

[13] Sohail S, Yu L, Bennett DA, Buchman AS, Lim ASP. Irregular 24-hour activity rhythms and the metabolic syndrome in older adults. Chronobiol Int. 2015;32:802–13. doi: 10.3109/07420528.2015.1041597 26061588 PMC4542004

[14] Zuurbier LA, Luik AI, Hofman A, Franco OH, Van Someren EJW, Tiemeier H. Fragmentation and stability of circadian activity rhythms predict mortality: the Rotterdam study. Am J Epidemiol. 2015;181:54–63. doi: 10.1093/aje/kwu245 25491893

[15] IBGE | Biblioteca | Detalhes | Censo demográfico: 2010: características da população e dos domicílios: resultados do universo [Internet]. [cited 2023 Nov 7]. https://biblioteca.ibge.gov.br/index.php/biblioteca-catalogo?view=detalhes&id=793

[16] Migueles JH, Rowlands AV, Huber F, Sabia S, van Hees VT. GGIR: A Research Community–Driven Open Source R Package for Generating Physical Activity and Sleep Outcomes From Multi-Day Raw Accelerometer Data. J Meas Phys Behav. 2019;2:188–96.

[17] van Hees VT, Gorzelniak L, Dean León EC, Eder M, Pias M, Taherian S, et al. Separating movement and gravity components in an acceleration signal and implications for the assessment of human daily physical activity. PloS One. 2013;8:e61691. doi: 10.1371/journal.pone.0061691 23626718 PMC3634007

[18] Van Someren EJ, Swaab DF, Colenda CC, Cohen W, McCall WV, Rosenquist PB. Bright light therapy: improved sensitivity to its effects on rest-activity rhythms in Alzheimer patients by application of nonparametric methods. Chronobiol Int. 1999;16:505–18. doi: 10.3109/07420529908998724 10442243

[19] van Hees VT, Sabia S, Jones SE, Wood AR, Anderson KN, Kivimäki M, et al. Estimating sleep parameters using an accelerometer without sleep diary. Sci Rep. 2018;8:12975. doi: 10.1038/s41598-018-31266-z 30154500 PMC6113241

[20] van Hees VT, Sabia S, Anderson KN, Denton SJ, Oliver J, Catt M, et al. A Novel, Open Access Method to Assess Sleep Duration Using a Wrist-Worn Accelerometer. PloS One. 2015;10:e0142533. doi: 10.1371/journal.pone.0142533 26569414 PMC4646630

[21] Plekhanova T, Rowlands AV, Davies MJ, Hall AP, Yates T, Edwardson CL. Validation of an automated sleep detection algorithm using data from multiple accelerometer brands. J Sleep Res. 2023;32:e13760. doi: 10.1111/jsr.13760 36317222

[22] Jones SE, van Hees VT, Mazzotti DR, Marques-Vidal P, Sabia S, van der Spek A, et al. Genetic studies of accelerometer-based sleep measures yield new insights into human sleep behaviour. Nat Commun. 2019;10:1585. doi: 10.1038/s41467-019-09576-1 30952852 PMC6451011

[23] Wendt A, da Silva ICM, Gonçalves H, Assunção MCF, Menezes AMB, Wehrmeister FC. Sleep parameters measured by accelerometry: descriptive analyses from the 22-year follow-up of the Pelotas 1993 birth cohort. Sleep Med. 2020;67:83–90. doi: 10.1016/j.sleep.2019.10.020 31918122

[24] Hildebrand M, VAN Hees VT, Hansen BH, Ekelund U. Age group comparability of raw accelerometer output from wrist- and hip-worn monitors. Med Sci Sports Exerc. 2014;46:1816–24. doi: 10.1249/MSS.0000000000000289 24887173

[25] Hildebrand M, Hansen BH, van Hees VT, Ekelund U. Evaluation of raw acceleration sedentary thresholds in children and adults. Scand J Med Sci Sports. 2017;27:1814–23. doi: 10.1111/sms.12795 27878845

[26] Barros AJD, Victora CG. [A nationwide wealth score based on the 2000 Brazilian demographic census]. Rev Saude Publica. 2005;39:523–9. 16113899 10.1590/s0034-89102005000400002

[27] Mander BA, Winer JR, Walker MP. Sleep and Human Aging. Neuron. 2017;94:19–36. doi: 10.1016/j.neuron.2017.02.004 28384471 PMC5810920

[28] Rogers-Soeder TS, Blackwell T, Yaffe K, Ancoli-Israel S, Redline S, Cauley JA, et al. Rest-Activity Rhythms and Cognitive Decline in Older Men: The Osteoporotic Fractures in Men Sleep Study. J Am Geriatr Soc. 2018;66:2136–43. doi: 10.1111/jgs.15555 30136716 PMC6235690

[29] Musiek ES, Bhimasani M, Zangrilli MA, Morris JC, Holtzman DM, Ju Y-ES. Circadian Rest-Activity Pattern Changes in Aging and Preclinical Alzheimer Disease. JAMA Neurol. 2018;75:582–90. doi: 10.1001/jamaneurol.2017.4719 29379963 PMC5885197

[30] 24-h Activity Rhythms and Health in Older Adults | Current Sleep Medicine Reports [Internet]. [cited 2023 Nov 7]. https://link.springer.com/article/10.1007/s40675-020-00170-2

[31] Martinez-Nicolas A, Madrid JA, García FJ, Campos M, Moreno-Casbas MT, Almaida-Pagán PF, et al. Circadian monitoring as an aging predictor. Sci Rep. 2018;8:15027. doi: 10.1038/s41598-018-33195-3 30301951 PMC6177481

[32] Luik AI, Zuurbier LA, Hofman A, Van Someren EJW, Tiemeier H. Stability and fragmentation of the activity rhythm across the sleep-wake cycle: the importance of age, lifestyle, and mental health. Chronobiol Int. 2013;30:1223–30. doi: 10.3109/07420528.2013.813528 23971909

[33] Kawakami F, Okamura H, Tamada Y, Maebayashi Y, Fukui K, Ibata Y. Loss of day-night differences in VIP mRNA levels in the suprachiasmatic nucleus of aged rats. Neurosci Lett. 1997;222:99–102. doi: 10.1016/s0304-3940(97)13355-9 9111738

[34] Roozendaal B, van Gool WA, Swaab DF, Hoogendijk JE, Mirmiran M. Changes in vasopressin cells of the rat suprachiasmatic nucleus with aging. Brain Res. 1987;409:259–64. doi: 10.1016/0006-8993(87)90710-4 3555704

[35] Watanabe A, Shibata S, Watanabe S. Circadian rhythm of spontaneous neuronal activity in the suprachiasmatic nucleus of old hamster in vitro. Brain Res. 1995;695:237–9. doi: 10.1016/0006-8993(95)00713-z 8556336

[36] Duffy JF, Zeitzer JM, Rimmer DW, Klerman EB, Dijk D-J, Czeisler CA. Peak of circadian melatonin rhythm occurs later within the sleep of older subjects. Am J Physiol Endocrinol Metab. 2002;282:E297–303. doi: 10.1152/ajpendo.00268.2001 11788360

[37] Walsh CM, Blackwell T, Tranah GJ, Stone KL, Ancoli-Israel S, Redline S, et al. Weaker circadian activity rhythms are associated with poorer executive function in older women. Sleep. 2014;37:2009–16. doi: 10.5665/sleep.4260 25337947 PMC4548515

[38] Gabriel BM, Zierath JR. Circadian rhythms and exercise—re-setting the clock in metabolic disease. Nat Rev Endocrinol. 2019;15:197–206. doi: 10.1038/s41574-018-0150-x 30655625

[39] Hower IM, Harper SA, Buford TW. Circadian Rhythms, Exercise, and Cardiovascular Health. J Circadian Rhythms. 2018;16:7. doi: 10.5334/jcr.164 30210567 PMC6083774

[40] Integration of short bouts of physical activity into organizational routine a systematic review of the literature—PubMed [Internet]. [cited 2023 Nov 7]. https://pubmed.ncbi.nlm.nih.gov/21146772/10.1016/j.amepre.2010.09.033 21146772

[41] Chen L-J, Hamer M, Lai Y-J, Huang B-H, Ku P-W, Stamatakis E. Can physical activity eliminate the mortality risk associated with poor sleep? A 15-year follow-up of 341,248 MJ Cohort participants. J Sport Health Sci. 2022;11:596–604. doi: 10.1016/j.jshs.2021.03.001 33713846 PMC9532590

[42] The circadian typology: the role of physical activity and melatonin [Internet]. [cited 2023 Nov 7]. https://ouci.dntb.gov.ua/en/works/4geMjQr9/

