# Supplementary material for: Is rest-activity rhythm prospectively associated with all-cause mortality in older people regardless of sleep and physical activity level? The ‘Como Vai?’ Cohort study
Source: PLoS One. 2024 Feb 16;19(2):e0298031. doi: 10.1371/journal.pone.0298031 (PMC10871497; doi:10.1371/journal.pone.0298031)
Supplement: S1 Table — Model 1: Crude analysis (exposure and outcome); Model 2: Model 1 + sex, age, socioeconomic status years of education; Model 3: Model 2 + current smoking status, morbidity score and number of medicines; Total sleep time, inactivity and MVPA expressed in blocks of 10 minutes. Overall PA expressed in mg. (PDF) [file pone.0298031.s001.pdf]

Tables S1A. Crude and adjusted association of total sleep time, overall physical activity, time spent in inactivity and moderate-to vigorous physical activity with all-cause mortality in older adults.

Rest-activity rhythm variables	Model 1		Model 2		Model 3	
	**HR (95%CI)**	**P value**	**HR (95%CI)**	**P value**	**HR (95%CI)**	**P value**
**Total sleep time**	1.05 (1.01; 1.09)	0.007	1.05 (1.02; 1.08)	0.002	1.05 (1.02; 1.09)	0.005
**Inactivity**	1.04 (1.02; 1.05)	<0.001	1.01 (0.99; 1.04)	0.173	1.01 (0.99; 1.03)	0.587
**Overall PA**	0.85 (0.82; 0.88)	<0.001	0.88 (0.84; 0.91)	<0.001	0.89 (0.85; 0.95)	<0.001
**MVPA**	0.27 (0.14; 0.55)	<0.001	0.35 (0.18; 0.67)	0.002	0.46 (0.25; 0.85)	0.014

Model 1: Crude analysis (exposure and outcome).

Model 2: Model 1 + sex, age, socioeconomic status years of education

Model 3: Model 2 + current smoking status, morbidity score and number of medicines

Total sleep time, inactivity and MVPA expressed in blocks of 10 minutes. Overall PA expressed in mg.
