# Supplementary material for: Is rest-activity rhythm prospectively associated with all-cause mortality in older people regardless of sleep and physical activity level? The ‘Como Vai?’ Cohort study
Source: PLoS One. 2024 Feb 16;19(2):e0298031. doi: 10.1371/journal.pone.0298031 (PMC10871497; doi:10.1371/journal.pone.0298031)
Supplement: S2 Table — Model 1: Crude analysis (exposure and outcome); Model 2: Model 1 + sex, age, socioeconomic status years of education; Model 3: Model 2 + current smoking status, morbidity score and number of medicines. (PDF) [file pone.0298031.s002.pdf]

Table S2A. Crude and adjusted association of intradaily variability and interdaily stability with all-cause mortality in older adults – excluding individuals who died in the period up to one year from the baseline.

	Model 1		Model 2		Model 3	
	**HR (95%CI)**	**P value**	**HR (95%CI)**	**P value**	**HR (95%CI)**	**P value**
**Intradaily variability (sd)**	1.40 (1.20; 1.64)	<0.001	1.29 (1.09; 1.52)	0.003	1.16 (0.94; 1.43)	0.164
**Interdaily stability (sd)**	0.57 (0.43; 0.77)	<0.001	0.63 (0.46; 0.86)	0.004	0.69 (0.49; 0.97)	0.033

Model 1: Crude analysis (exposure and outcome).

Model 2: Model 1 + sex, age, socioeconomic status years of education

Model 3: Model 2 + current smoking status, morbidity score and number of medicines
