# Supplementary material for: Is rest-activity rhythm prospectively associated with all-cause mortality in older people regardless of sleep and physical activity level? The ‘Como Vai?’ Cohort study
Source: PLoS One. 2024 Feb 16;19(2):e0298031. doi: 10.1371/journal.pone.0298031 (PMC10871497; doi:10.1371/journal.pone.0298031)
Supplement: S3 Table — Model 3: sex, age, socioeconomic status years of education, current smoking status, morbidity score and number of medicines; Model 4: Model 3 + adjustment for total sleep time; Model 5: Model 3 + adjustment for inactivity; Model 6: Model 3 + adjustment for overall physical activity; Model 7: Model 3 + adjustment for MVPA. (PDF) [file pone.0298031.s003.pdf]

Table S3A. Association of intradaily variability and interdaily stability and all-cause mortality in older adults adjusted for intensity levels – excluding individuals who died in the period up to one year from the baseline.

	Model 4		Model 5		Model 6		Model 7	
	**HR (95%CI)**	**P value**	**HR (95%CI)**	**P value**	**HR (95%CI)**	**P value**	**HR (95%CI)**	**P value**
**Intradaily variability (sd)**	1.17 (0.96; 1.43)	0.116	1.13 (0.91; 1.41)	0.263	0.92 (0.74; 1.14)	0.431	1.00 (0.78; 1.29)	0.977
**Interdaily stability (sd)**	0.67 (0.48; 0.93)	0.019	0.70 (0.47; 1.03)	0.068	1.07 (0.73; 1.56)	0.743	0.76 (0.55; 1.05)	0.091

Model 3: sex, age, socioeconomic status years of education, current smoking status, morbidity score and number of medicines

Model 4: Model 3 + adjustment for total sleep time

Model 5: Model 3 + adjustment for inactivity

Model 6: Model 3 + adjustment for overall physical activity

Model 7: Model 3 + adjustment for MVPA
